# Supplementary material for: Migratory network reveals unique spatial-temporal migration dynamics of Dunlin subspecies along the East Asian-Australasian Flyway
Source: PLoS One. 2022 Aug 4;17(8):e0270957. doi: 10.1371/journal.pone.0270957 (PMC9352067; doi:10.1371/journal.pone.0270957)
Supplement: S1 Appendix — Annotated R code of steps taken to generate geolocator-derived stationary estimates and refine Dunlin migration tracks along the East Asian-Australasian Flyway. (HTML) [file pone.0270957.s001.html]

## S1 APPENDIX: Light-level geolocator analyses

####

#### Lagassé et al. Migratory network reveals unique spatial-temporal migration dynamics of Dunlin subspecies along the EAAF.

#### 

The following code borrows from:

Bridge, E., S. Lisovski, E. Rakhimberdiev, and M. Hallworth (2016). Geolocation analysis with Open Source Tools. North American Ornithological Congress, Washington D.C. [link] (https://github.com/Eli-S-Bridge/NAOC\_Geos\_2016)

```
rm(list=ls())
z<-c("TwGeos", "FLightR", "ggplot2","maptools","raster", "lubridate")
lapply(z, library, character.only=TRUE)
setwd("~/Desktop/geo.supplement")
```

####

## Generating stationary estimates

#### 

##### (1) Upload light file, (2) crop deployment period, (3) identify twilights

```
#1
raw.lig<-readLig("arct_0711.lig")

#2
depl_substart<-'2010-06-19' 
depl_subend<-'2011-07-16' 
depl.lig<-subset(raw.lig,Date>=depl_substart & Date<=depl_subend, select=Date:Light)

#3
threshold<-5
seed <- as.POSIXct("2010-11-01 16:00", origin  = "1970-01-01", tz = "GMT")
depl.twl<-findTwilights(depl.lig, threshold=threshold, include=seed)

lightImage(depl.lig, dt=120, offset=03, zlim=c(0, 64))
tsimagePoints(depl.twl$Twilight, offset=03, pch = 16, cex = 0.5,col = ifelse(depl.twl$Rise, "dodgerblue", "firebrick"))
```

#####

##### Identify and discard incorrect twilight assignments

Incorrect twilight assignments occur from periodic shading of the light sensor (e.g., if an adult roosted with the geolocator tucked among its body feathers). 1: Identify twilights that are greater than 45 minutes different than twilights that occurred in the surrounding 4 days (2 days before and 2 days after). 2: If neighboring twilights are within 15 minutes of each other, adjust outlying twilight between neighboring twilights. 3: If neighboring twilights are not within 15 minutes of each other, discard outlying twilight. 4: Remove discarded twilights.

```
#1,2,3
depl.edit<-twilightEdit(depl.twl, offset=03, window= 4, stationary.mins = 15, outlier.mins= 45)
```

```
#4
depl.edit <- subset(depl.edit, Deleted == FALSE)
```

#####

##### Transform twilight times from BAStag to TAGS format to use in FLightR

```
#remove column 'Julian'
depl.lig<- subset(depl.lig, select = -c(Julian))

##Transform twilight times from BAStag to TAGS format to use in FLightR
BAStag2TAGS(depl.lig, depl.edit, threshold=threshold, filename="depl.TAGS.csv")
```

```
## NULL
```

```
#upload from .csv
depl.TAGS<-get.tags.data("depl.TAGS.csv")
```

```
## Detected mk tag
## tag saved data every 120 seconds, and is assumed to measure data every 60 seconds, and write down max
```

#####

##### Rooftop calibration

We used a pre-deployment rooftop calibration for tags that were deployed at breeding sites north of 66.7˚N where 24-hour sunlight precluded the identification of twilights in the light intensity data.

```
#subset rooftop calibration period
roof_substart<-'2010-05-16' 
roof_subend<-'2010-05-21' 
roof.cal<-subset(raw.lig,Date>=roof_substart & Date<=roof_subend, select=Date:Light)

#assign twilights
seed <- as.POSIXct("2010-05-19 10:00", origin  = "1970-01-01", tz = "GMT")
roof.twl<-findTwilights(roof.cal, threshold=threshold, include=seed)

#remove column 'Julian', get twl into format for BAStag2TAGS() 
roof.cal<- subset(roof.cal, select = -c(Julian))
roof.edit<-twilightEdit(roof.twl, offset=6, window= 1)

#transform to TAGS format
BAStag2TAGS(roof.cal, roof.edit, threshold=threshold, filename="roof.TAGS.csv")

#upload to use in FLightR
roof.TAGS<-get.tags.data("roof.TAGS.csv")

#assign period and location of calibration
Calibration.periods<-data.frame(
  calibration.start=as.POSIXct(c("2010-05-17")),
  calibration.stop=as.POSIXct(c("2010-05-20")),
  lon=c(-150.09), lat=c(61.41)) 

#generate calibration parameters
roof.cal<-make.calibration(roof.TAGS, Calibration.periods)
```

#####

##### Assign spatial extent and behavior mask

We parameterized the movement model so that location estimates over land or over ocean were equally likely if ambient light levels indicated an individual was in a migratory state, but were weighted toward land if light levels indicated an individual was in a sedentary state.

```
Grid<-make.grid(left=80, bottom=0, right=-140, top=75,
                distance.from.land.allowed.to.use=c(-Inf, Inf),
                distance.from.land.allowed.to.stay=c(-2500, 1),
                probability.of.staying = 0.5)
```

#####

##### Create prerun object

```
all.in.roof.arct_0711<-make.prerun.object(depl.TAGS, Grid, 
                           start=c(-154.73, 70.55),
                           Calibration=roof.cal)
```

#####

##### Run particle filter

```
nParticles<-1e6
out.data<-run.particle.filter(all.in.roof.arct_0711,
            nParticles=nParticles, known.last=FALSE,
            check.outliers=TRUE)
```

#####

##### Test plot

```
plot_lon_lat(out.data, scheme=c("vertical"))
```

#####

##### Estimate longitude and latitiude of locations where bird was stationary 2+ days, and their arrival and departure dates

```
stat.data<-stationary.migration.summary(out.data, prob.cutoff=0.25, min.stay = 4)
```

```
#create data.frame of stationary locations
roof.loc<-as.data.frame(cbind(stat.data$Stationary.periods$Medianlon,
                stat.data$Stationary.periods$Medianlat,
                stat.data$Potential_stat_periods$start,
                stat.data$Potential_stat_periods$end,
                stat.data$Potential_stat_periods$Duration,
                stat.data$Stationary.periods$FstQu.lon,
                stat.data$Stationary.periods$FstQu.lat,
                stat.data$Stationary.periods$TrdQu.lon,
                stat.data$Stationary.periods$TrdQu.lat)) 

#rename columns
colnames(roof.loc)<-c("lon","lat","arr","dep","dur","FstQu.lon","FstQu.lat","TrdQu.lon","TrdQu.lat")

#add arrival/departure dates and format as POSIXct
for(i in 1:dim(roof.loc)[1]){
  if (roof.loc$arr > (0)){
    roof.loc$arr[i]<-out.data$Results$Movement.results$Real.time[(roof.loc$arr[i])]
    roof.loc$dep[i]<-out.data$Results$Movement.results$Real.time[(roof.loc$dep[i])]}}

roof.loc$arr<-as.POSIXct(roof.loc$arr, origin = "1970-01-01", tz="GMT")
roof.loc$dep<-as.POSIXct(roof.loc$dep, origin = "1970-01-01", tz="GMT")

#calculate number of days at each site
for (i in 1:dim(roof.loc)[1]){
  roof.loc$dur[i]<-round((difftime(roof.loc$dep[i],roof.loc$arr[i],units=c("days"))), digits=0)}

#add column 'tag' as a unique id
roof.loc$tag<-"arct_0711"
```

##### Plot stationary estimates

```
#transform locations so path crosses date line
west2east <- function(neg.long) {
  diff.long <- (180+neg.long)
  west.long <- (diff.long+180)
  return(west.long)}

#calculate non-negative lon/lat for all estimates
for (i in 1:dim(roof.loc)[1]) {
  if (roof.loc$lon[i] < 0)
  {roof.loc$lon[i]<-west2east(roof.loc$lon[i])}
  if (roof.loc$FstQu.lon[i] < 0)
  {roof.loc$FstQu.lon[i]<-west2east(roof.loc$FstQu.lon[i])}
  if (roof.loc$TrdQu.lon[i] < 0) 
  {roof.loc$TrdQu.lon[i]<-west2east(roof.loc$TrdQu.lon[i])}}

#add month column for color scale
roof.loc$month<-month(roof.loc$arr)
```

```
#create basemap
data(wrld_simpl)
wrld<- wrld_simpl[!(wrld_simpl$NAME %in% c("Greenland","Antarctica")),]

band.aid<-data.frame(x=c(180.075, 179.85, 179.85, 180.075, 180.075, 179.85, 180.075, 180.075, 179.85, 179.85), y=c(68.8, 68.9, 65.15, 65.295, 68.8, 71.07, 71.07, 71.42, 71.42, 71.07))
band.aid$id<-c(1,1,1,1,1,2,2,2,2,2)

base.map<- ggplot(wrld,aes(x=long,y=lat,group=group))+
  geom_polygon(fill="grey85",color="black")+
  coord_map(projection="azequalarea", orientation=c(36,127,0), xlim=c(106,217), ylim=c(16,75))+
  theme_bw()+
  scale_x_continuous("", breaks = c(125, 150, 175),
                     labels = c("125°E", "150°E", "175°E"))+
  scale_y_continuous("", breaks = c(20, 40, 60),
                     labels = c("20°N","40°N","60°N"))+
  theme(axis.title.x=element_blank()) + theme(axis.title.y=element_blank())+
  geom_polygon(data=band.aid,aes(x=x, y=y, group=id), fill="grey85", color="grey85")
```

```
## Regions defined for each Polygons
```

```
###
my_title <- expression(paste("arct_0711; stationary estimates (rooftop calibration)"))
###
base.map+geom_path(data=roof.loc, show.legend=FALSE, colour="black", size=1, alpha=0.85, aes(x=lon, y=lat, group = tag))+
  geom_point(data=roof.loc, alpha=1, shape = 19, aes(x=lon, y=lat, group = tag, size=dur, colour=month))+
  scale_size(limits = c(2,300))+scale_color_gradientn(colours = rainbow(4), limits=c(1, 12))+
  labs(colour="month",size="days at site")+
  theme(legend.box="vertical",legend.position = c(0.9, 0.5))+
  labs(title=my_title)
```

#####

##### Re-calibration

After generating an initial migration track, we re-calibrated each geolocator using the median latitude, longitude, and light level data while the bird was at its longest estimated stationary site (typically during the boreal winter). We chose to re-calibrate because calibration parameters from a nonbreeding site are better at accounting for environmentally- and behaviorally-induced noise in light-level recordings at nonbreeding sites than calibration parameters from a rooftop calibration, or while a bird was on its breeding grounds

```
#longest stationary period
roof.loc[24,1:4]
```

```
##         lon  lat                 arr                 dep
## 24 121.9753 24.3 2011-02-14 22:17:22 2011-03-20 21:45:48
```

```
#assign time and location for calibration
Calibration.periods<-data.frame(
  calibration.start=as.POSIXct(c("2011-2-15")),
  calibration.stop=as.POSIXct(c("2011-3-19")),
  lon=c(121.9753), lat=c(24.3))

#run re-calibration parameters
re.cal<-make.calibration(depl.TAGS, Calibration.periods, model.ageing=FALSE, plot.final=FALSE)
```

#####

##### Re-calibration prerun object & particle filter

```
all.in.re.arct_0711<-make.prerun.object(depl.TAGS, Grid, 
                           start=c(-154.73, 70.55), 
                           Calibration=re.cal)

nParticles<-1e6
re.out.data<-run.particle.filter(all.in.re.arct_0711,
            nParticles=nParticles, known.last=FALSE,
            check.outliers=TRUE)
```

#####

##### Test plot

```
plot_lon_lat(re.out.data, scheme=c("vertical"))
```

#####

##### Re-estimate longitude and latitiude of locations where bird was stationary 2+ days, and their arrival and departure dates

```
restat.data<-stationary.migration.summary(re.out.data, prob.cutoff=0.25, min.stay = 4)
```

```
#create data.frame of stationary locations
re.loc<-as.data.frame(cbind(restat.data$Stationary.periods$Medianlon,
                  restat.data$Stationary.periods$Medianlat,
                  restat.data$Potential_stat_periods$start,
                  restat.data$Potential_stat_periods$end,
                  restat.data$Potential_stat_periods$Duration,
                  restat.data$Stationary.periods$FstQu.lon,
                  restat.data$Stationary.periods$FstQu.lat,
                  restat.data$Stationary.periods$TrdQu.lon,
                  restat.data$Stationary.periods$TrdQu.lat)) 

re.loc[1,3]<-1

#rename columns
colnames(re.loc)<-c("lon","lat","arr","dep","dur","FstQu.lon","FstQu.lat","TrdQu.lon","TrdQu.lat")

#add arrival/departure dates and format as POSIXct
for(i in 1:dim(re.loc)[1]){
  if (re.loc$arr > (0)){
    re.loc$arr[i]<-re.out.data$Results$Movement.results$Real.time[(re.loc$arr[i])]
    re.loc$dep[i]<-re.out.data$Results$Movement.results$Real.time[(re.loc$dep[i])]}}

re.loc$arr<-as.POSIXct(re.loc$arr, origin = "1970-01-01", tz="GMT")
re.loc$dep<-as.POSIXct(re.loc$dep, origin = "1970-01-01", tz="GMT")

#calculate number of days at each site
for (i in 1:dim(re.loc)[1]){
  re.loc$dur[i]<-round((difftime(re.loc$dep[i],re.loc$arr[i],units=c("days"))), digits=0)}

#add column 'tag' as a unique id
re.loc$tag<-"arct_0711"
```

##### Plot stationary estimates

```
#calculate non-negative lon/lat for all estimates
for (i in 1:dim(re.loc)[1]) {
  if (re.loc$lon[i] < 0)
  {re.loc$lon[i]<-west2east(re.loc$lon[i])}
  if (re.loc$FstQu.lon[i] < 0)
  {re.loc$FstQu.lon[i]<-west2east(re.loc$FstQu.lon[i])}
  if (re.loc$TrdQu.lon[i] < 0) 
  {re.loc$TrdQu.lon[i]<-west2east(re.loc$TrdQu.lon[i])}}

#add month column for color scale
re.loc$month<-month(re.loc$arr)
```

```
###
my_title <- expression(paste("arct_0711; stationary estimates (re-calibration)"))
#add points and lines
base.map+geom_path(data=re.loc, show.legend=FALSE, colour="black", size=1, alpha=0.85, aes(x=lon, y=lat, group = tag))+
  geom_point(data=re.loc, alpha=1, shape = 19, aes(x=lon, y=lat, group = tag, size=dur, colour=month))+
  scale_size(limits = c(2,300))+scale_color_gradientn(colours = rainbow(4), limits=c(1, 12))+
  labs(colour="month",size="days at site")+
  theme(legend.box="vertical",legend.position = c(0.9, 0.5))+
  labs(title=my_title)
```

####

## Refining migration tracks

#### 

##### Merge stationary estimates < 250 km apart

Because of the general inaccuracy of solar geolocation, we merged all sequential stationary estimates that were < 250 km apart by averaging geographic coordinates and combining arrival/departure dates. This distance is a conservative buffer for the geographic resolution of stationary estimates returned by FLightR and functions to aggregate routine movements that may occur during a stationary period (e.g., daily movements between roosting and foraging sites).

```
merge.tracks <- function(df, na.rm = TRUE, ...){
  
  #data.frame for compilation
  pop.merge<-df[1,]
  
  #create list of geolocator tracks in dataframe 
  tag_id <- unique(df$tag)
  
  # create for loop to merge each tag 
  for (i in seq_along(tag_id)) { 
    
    #subset each tag in succession 
    bird<-subset(df, df$tag==tag_id[i])
    bird$merge.with<-99
    
    #calculate distance matrix
    repeat {bird<-subset(bird, merge.with > 98)
    a<-cbind(bird$lon,bird$lat)
    b<-pointDistance(a,a, lonlat=TRUE, allpairs=TRUE)
    
    #do the merging
    for (i in 1:dim(bird)[1]){
      tryCatch({
        if (b[(i+1),i] < 250000.0)
        {bird$merge.with[i] <- i+1
        bird$dur[i+1] <- (bird$dur[i]+bird$dur[i+1])
        bird$arr[i+1] <- bird$arr[i]
        bird$lon[i+1] <- mean(c(bird[(i:(i+1)),1]))
        bird$lat[i+1] <- mean(c(bird[(i:(i+1)),2]))
        bird$FstQu.lon[i+1] <- mean(c(bird[(i:(i+1)),6]))
        bird$FstQu.lat[i+1] <- mean(c(bird[(i:(i+1)),7]))
        bird$TrdQu.lon[i+1] <- mean(c(bird[(i:(i+1)),8]))
        bird$TrdQu.lat[i+1] <- mean(c(bird[(i:(i+1)),9]))}
      }, error=function(e){cat("ERROR :",conditionMessage(e), "\n")})
      if (bird$merge.with[i] < 99)
      {break}}
    
    if (min(bird$merge.with) > 98)
    {break}
    }
    
    bird<-bird[,-12]
    pop.merge<-rbind(pop.merge,bird)
  }
  
  #remove filler
  pop.merge<-pop.merge[-1,]
  
  #add month
  pop.merge$month<-month(pop.merge$arr)
  
  #stopover periods
  for (i in 1:dim(pop.merge)[1]){
    pop.merge$dur[i]<-round((difftime(pop.merge$dep[i], pop.merge$arr[i], units=c("days"))), digits=0)}
  
  return(pop.merge)
}

merge.0711<-merge.tracks(re.loc)
```

```
###
my_title <- expression(paste("arct_0711; merge stationary estimates < 250km apart"))
#add points and lines
base.map+geom_path(data=merge.0711, show.legend=FALSE, colour="black", size=1, alpha=0.85, aes(x=lon, y=lat, group = tag))+
  geom_point(data=merge.0711, alpha=1, shape = 19, aes(x=lon, y=lat, group = tag, size=dur, colour=month))+
  scale_size(limits = c(2,300))+scale_color_gradientn(colours = rainbow(4), limits=c(1, 12))+
  theme(axis.title.x=element_blank(), axis.text.x=element_blank(), axis.ticks.x=element_blank())+
  theme(axis.title.y=element_blank(), axis.text.y=element_blank(), axis.ticks.y=element_blank())+
  labs(colour="month",size="days at site")+
  theme(legend.box="vertical",legend.position = c(0.9, 0.5))+
  labs(title=my_title)
```

#####

##### Remove points > 66.7˚N & < 250 km from breeding site

We also discarded stationary estimates prior to a bird travelling > 250 km from their known breeding site (i.e., capture/re-capture site) and, because solar geolocation performs poorly at high latitudes, we discarded stationary estimates prior to a bird travelling south of 66.7˚N. We then connected the first and last stationary estimates with the known breeding site.

```
#remove location > 66.7 N and < 250 km from breeding site
merge.0711<-merge.0711[-1,]

#add breeding location
df<-data.frame("205.27","70.55","2010-06-01 00:00:00","2010-06-01 00:00:00","0","205.27","70.55","205.27","70.55","arct_0711","6")
names(df)<-c("lon","lat","arr","dep","dur","FstQu.lon","FstQu.lat","TrdQu.lon","TrdQu.lat","tag","month")

df$lon<-as.numeric(df$lon)
df$lat<-as.numeric(df$lat)
df$arr<-as.POSIXct(df$arr, origin = "1970-01-01", tz="GMT")
df$dep<-as.POSIXct(df$dep, origin = "1970-01-01", tz="GMT")
df$dur<-as.numeric(df$dur)
df$FstQu.lon<-as.numeric(df$FstQu.lon)
df$FstQu.lat<-as.numeric(df$FstQu.lat)
df$TrdQu.lon<-as.numeric(df$TrdQu.lon)
df$TrdQu.lat<-as.numeric(df$TrdQu.lat)
df$month<-as.numeric(df$month)

merge.0711<-rbind(merge.0711,df)
merge.0711<-rbind(df,merge.0711)
```

```
###
my_title <- expression(paste("arct_0711; remove stationary estimates > 66.7˚N & < 250km"))
#add points and lines
base.map+geom_path(data=merge.0711, show.legend=FALSE, colour="black", size=1, alpha=0.85, aes(x=lon, y=lat, group = tag))+
  geom_point(data=merge.0711, alpha=1, shape = 19, aes(x=lon, y=lat, group = tag, size=dur, colour=month))+
  scale_size(limits = c(2,300))+scale_color_gradientn(colours = rainbow(4), limits=c(1, 12))+
  geom_point(data=merge.0711,aes(x=merge.0711[1,1], y=merge.0711[1,2], group = tag), size=3, colour="black", fill="ivory", shape = 25, stroke=1.5,
             show.legend=FALSE)+
  theme(axis.title.x=element_blank(), axis.text.x=element_blank(), axis.ticks.x=element_blank())+
  theme(axis.title.y=element_blank(), axis.text.y=element_blank(), axis.ticks.y=element_blank())+
  labs(colour="month",size="days at site")+
  theme(legend.box="vertical",legend.position = c(0.9, 0.5))+
  labs(title=my_title)
```

#####

##### Smooth stationary estimates with a turning angle < 60˚

Finally, because FLightR can generate erratic stationary estimates due to noisy light-level data, we discarded stationary estimates that had a turning angle of < 60˚ (i.e., locations comprising an angle of < 60˚ between their prior and subsequent stationary estimate) but combined their arrival/departure date with their nearest neighbor. We did not follow this procedure for the stationary estimate that was farthest from the breeding site and had a stationary period > 41 days. This approach assumes that an individual migrated without reversing direction until they departed their most distant winter site to migrate north to breed. It also assumes that an individual would stop at its farthest winter site for > 41 days, a minimum winter duration supported by prior Dunlin tracking studies and repeat band resightings on the EAAF. Although these assumptions might not be fully met (e.g., a bird could exhibit north-south movements during migration or winter), spatial inaccuracies in estimating latitude preclude finer resolving of the tracks.

```
#identify south and north segments
merge.0711$south<-c("TRUE","TRUE","TRUE","TRUE","TRUE","TRUE","TRUE","TRUE","TRUE","TRUE","FALSE","FALSE","FALSE","FALSE","FALSE")
merge.0711$north<-c("FALSE","FALSE","FALSE","FALSE","FALSE","FALSE","FALSE","FALSE","FALSE","TRUE","TRUE","TRUE","TRUE","TRUE","TRUE")

smooth.tracks <- function(df, na.rm = TRUE, ...){
  
  #function that calculates angle
  #(https://stackoverflow.com/questions/11184381)
  trackAngle <- function(xy) {
    angles <- abs(c(trackAzimuth(xy), 0) -
                    c(0, rev(trackAzimuth(xy[nrow(xy):1, ]))))
    angles <- ifelse(angles > 180, 360 - angles, angles)
    angles[is.na(angles)] <- 180
    angles[-c(1, length(angles))]}
  
  #data.frame for compilation
  pop.smooth<-df[1,]
  
  #create list of geolocator tracks in dataframe 
  tag_id <- unique(df$tag)
  
  # create for loop to smooth each tag 
  for (i in seq_along(tag_id)) { 
    
    #separate south and north
    #create logical outlier column & col merge.with
    bird<-subset(df, df$tag==tag_id[i])
    bird$outlier[1:dim(bird)[1]] = FALSE
    bird$merge.with[1:dim(bird)[1]] <- 99
    south<-subset(bird, bird$south==TRUE)
    north<-subset(bird, bird$north==TRUE)
    north<-north[-1,]
    
    
    repeat {
      #remove outlier
      south<-subset(south, outlier==FALSE)
      #calculate angle
      south_angle<-data.matrix(south[1:2])
      south_angle<-trackAngle(south_angle)
      south_angle<-as.data.frame(south_angle)
      south$angle[1:dim(south)[1]]<-180
      south$angle[2:(dim(south)[1]-1)]<-south_angle$south_angle
      #calculate distance matrix
      a<-cbind(south$lon,south$lat)
      b<-pointDistance(a,a, lonlat=TRUE, allpairs=TRUE)
      
      for (j in 1:dim(south)[1]) {
        if (south$angle[j] < 60){
          south$outlier[j] = TRUE
          b[j,] <- 999999999
          south$merge.with[j] <- which(b[,j]==min(b[((j-1):(j+1)),j]))
          south$arr[south$merge.with[j]] <- pmin(south$arr[j],
                                                     south$arr[south$merge.with[j]])
          south$dep[south$merge.with[j]] <- pmax(south$dep[j],
                                                       south$dep[south$merge.with[j]])
        }
        
        if (south$merge.with[j] < 99)  
        {break}}
      
      if (min(south$angle) >= 60)
      {break}
    }
    
    
    #again for north migration
    repeat {south<-south[,-16]
    if (dim(south)[2] < 16)
    {break}}
    whole.again<-rbind(south,north)
    south.2<-south[-dim(south)[1],]
    north.2<-subset(whole.again, north=="TRUE")
    
    repeat {
      #remove outlier
      north.2<-subset(north.2, outlier==FALSE)
      #calculate angle
      north.2_angle<-data.matrix(north.2[1:2])
      north.2_angle<-trackAngle(north.2_angle)
      north.2_angle<-as.data.frame(north.2_angle)
      north.2$angle[1:dim(north.2)[1]]<-180
      north.2$angle[2:(dim(north.2)[1]-1)]<-north.2_angle$north.2_angle
      #calculate distance matrix
      a<-cbind(north.2$lon,north.2$lat)
      b<-pointDistance(a,a, lonlat=TRUE, allpairs=TRUE)
      
      for (j in 1:dim(north.2)[1]) {
        if (north.2$angle[j] < 60){
          north.2$outlier[j] = TRUE
          b[j,] <- 999999999
          north.2$merge.with[j] <- which(b[,j]==min(b[((j-1):(j+1)),j]))
          north.2$arr[north.2$merge.with[j]] <- pmin(north.2$arr[j],
                                                         north.2$arr[north.2$merge.with[j]])
          north.2$dep[north.2$merge.with[j]] <- pmax(north.2$dep[j],
                                                           north.2$dep[north.2$merge.with[j]])
        }
        
        if (north.2$merge.with[j] < 99)  
        {break}}
      
      if (min(north.2$angle) >= 60)
      {break}
    }
    
    
    #in the darkness bind them
    repeat {north.2<-north.2[,-16]
    if (dim(north.2)[2] < 16)
    {break}}
    smooth.again<-rbind(south.2,north.2)
    
    repeat {smooth.again<-smooth.again[,-14]
    if (dim(smooth.again)[2] < 14)
    {break}}
    
    #collate
    pop.smooth<-rbind(pop.smooth,smooth.again)
    
  }
  
  pop.smooth<-pop.smooth[-1,]
  
  #add month
  pop.smooth$month<-month(pop.smooth$arr)
  
  #stopover periods
  for (i in 1:dim(pop.smooth)[1]){
    pop.smooth$dur[i]<-round((difftime(pop.smooth$dep[i], pop.smooth$arr[i], units=c("days"))), digits=0)}
  
  return(pop.smooth[,1:11])
  
}

#RUN!
smooth.0711<-smooth.tracks(merge.0711)
```

```
###
my_title <- expression(paste("arct_0711; smooth stationary estimates w/turning angle < 60˚"))
#add points and lines
base.map+geom_path(data=smooth.0711, show.legend=FALSE, colour="black", size=1, alpha=0.85, aes(x=lon, y=lat, group = tag))+
  geom_point(data=smooth.0711, alpha=1, shape = 19, aes(x=lon, y=lat, group = tag, size=dur, colour=month))+
  scale_size(limits = c(2,300))+scale_color_gradientn(colours = rainbow(4), limits=c(1, 12))+
  geom_point(data=smooth.0711,aes(x=smooth.0711[1,1], y=smooth.0711[1,2], group = tag), size=3, colour="black", fill="ivory", shape = 25, stroke=1.5,
             show.legend=FALSE)+
  theme(axis.title.x=element_blank(), axis.text.x=element_blank(), axis.ticks.x=element_blank())+
  theme(axis.title.y=element_blank(), axis.text.y=element_blank(), axis.ticks.y=element_blank())+
  labs(colour="month",size="days at site")+
  theme(legend.box="vertical",legend.position = c(0.9, 0.5))+
  labs(title=my_title)
```

#####

##### Final check

After smoothing, merge any sequential stationary estimates < 250 km apart and discard stationary estimates prior to a bird travelling south of 66.7˚N.

```
fin.0711<-merge.tracks(smooth.0711)
fin.0711<-fin.0711[-2,]
```

```
###
my_title <- expression(paste("arct_0711; final stationary estimates"))
#add points and lines
base.map+geom_path(data=fin.0711, show.legend=FALSE, colour="black", size=1, alpha=0.85, aes(x=lon, y=lat, group = tag))+
  geom_point(data=fin.0711, alpha=1, shape = 19, aes(x=lon, y=lat, group = tag, size=dur, colour=month))+
  scale_size(limits = c(2,300))+scale_color_gradientn(colours = rainbow(4), limits=c(1, 12))+
  geom_point(data=fin.0711,aes(x=fin.0711[1,1], y=fin.0711[1,2], group = tag), size=3, colour="black", fill="ivory", shape = 25, stroke=1.5,
             show.legend=FALSE)+
  theme(axis.title.x=element_blank(), axis.text.x=element_blank(), axis.ticks.x=element_blank())+
  theme(axis.title.y=element_blank(), axis.text.y=element_blank(), axis.ticks.y=element_blank())+
  labs(colour="month",size="days at site")+
  theme(legend.box="vertical",legend.position = c(0.9, 0.5))+
  labs(title=my_title)
```
