# Supplementary material for: Migratory network reveals unique spatial-temporal migration dynamics of Dunlin subspecies along the East Asian-Australasian Flyway
Source: PLoS One. 2022 Aug 4;17(8):e0270957. doi: 10.1371/journal.pone.0270957 (PMC9352067; doi:10.1371/journal.pone.0270957)
Supplement: S1 Table — (PDF) [file pone.0270957.s003.pdf]

S1 Table. South migration timing of Dunlin subspecies by migration region. Reported is the interquartile range. See Figure 2 for the location of each region.

	C. a. kistchinki **(*n*=5)**		***C. a. sakhalina*** **(*n*=26)**		***C. a. arcticola*** **(*n*=52)**	
**Region**	**Arrival**	**Departure**	**Arrival**	**Departure**	**Arrival**	**Departure**
R 25	-	-	-	-	22 Aug–5 Sep	8 Oct–14 Oct
R 24	-	-	-	-	22 Aug–1 Sep	5 Sep–8 Oct
R 23	-	-	9 Aug–26 Aug	17 Aug–30 Aug	7 Sep–22 Sep	18 Sep–10 Oct
R 22	-	-	12 Aug–27 Aug	15 Aug–30 Aug	20 Sep–7 Oct	7 Oct–14 Oct
R 21	-	-	22 Aug–28 Aug	26 Aug–4 Sep	23 Sep–12 Oct	4 Oct–17 Oct
R 20	-	-	13 Aug–23 Aug	23 Aug–29 Aug	20 Sep–23 Sep	30 Sep–9 Oct
R 19	-	-	23 Aug–30 Aug	28 Aug–8 Sep	23 Sep–28 Sep	1 Oct–16 Oct
R 18	-	-	19 Sep–2 Oct	5 Oct–14 Oct	11 Oct–17 Oct	18 Oct–26 Oct
R 17	-	-	13 Aug–22 Aug	15 Aug–24 Aug	15 Oct	18 Oct
R 16	-	-	16 Aug–27 Aug	23 Aug–1 Sep	20 Sep–7 Oct	5 Oct–22 Oct
R 15	30 Jun–10 Jul	9 Jul–24 Jul	28 Aug–6 Sep	7 Sep–17 Sep	10 Oct–19 Oct	22 Oct–29 Oct
R 14	-	-	27 Aug–2 Sep	8 Sep–16 Sep	16 Oct–22 Oct	30 Oct–4 Nov
