# Supplementary figures and images for: Migratory network reveals unique spatial-temporal migration dynamics of Dunlin subspecies along the East Asian-Australasian Flyway
Source: PLoS One. 2022 Aug 4;17(8):e0270957. doi: 10.1371/journal.pone.0270957 (PMC9352067; doi:10.1371/journal.pone.0270957)

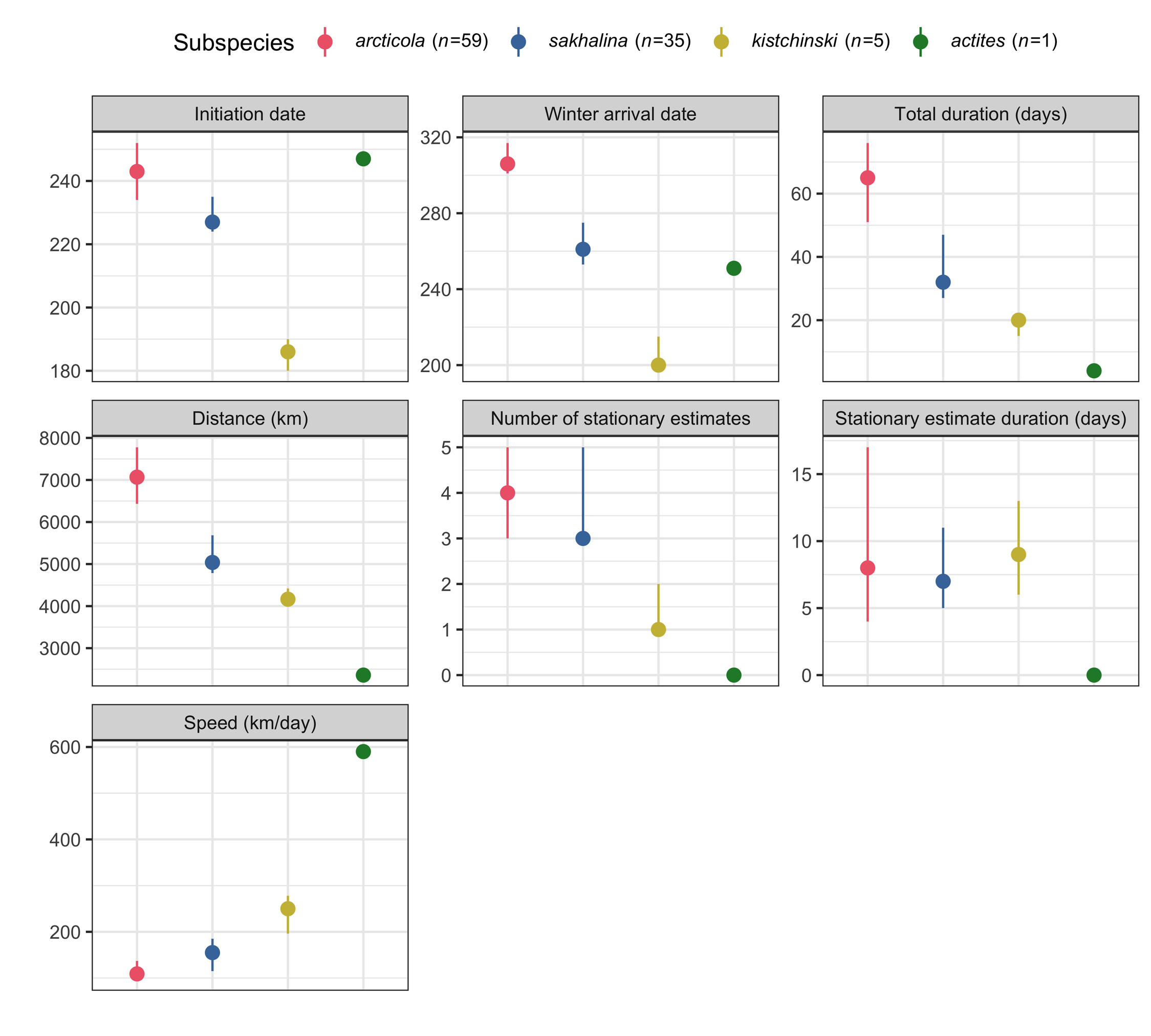

Supplement: S1 Fig — Reported is the median value and interquartile range. (TIF) [file pone.0270957.s004.tif]

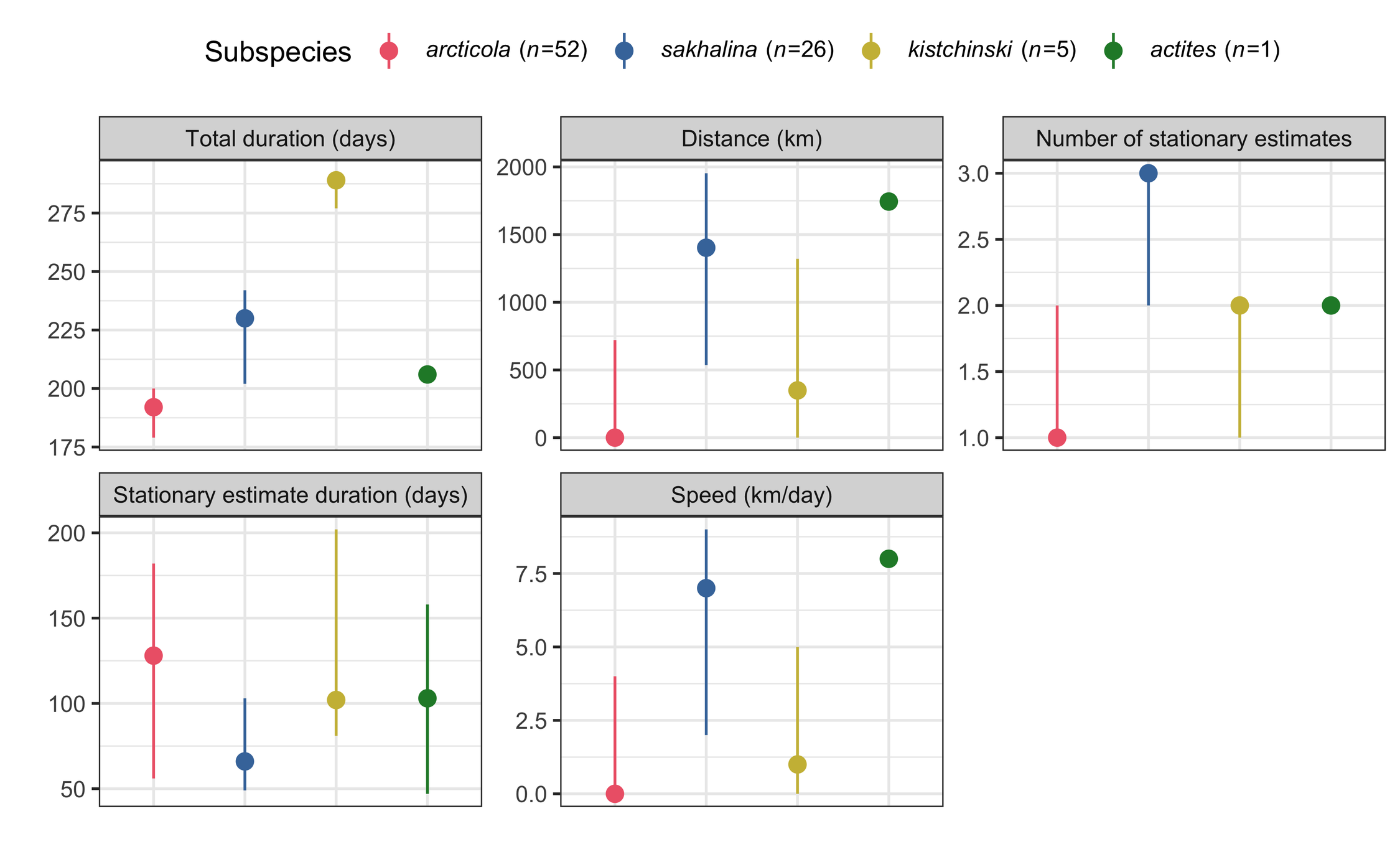

Supplement: S2 Fig — Reported is the median value and interquartile range. (TIF) [file pone.0270957.s005.tif]

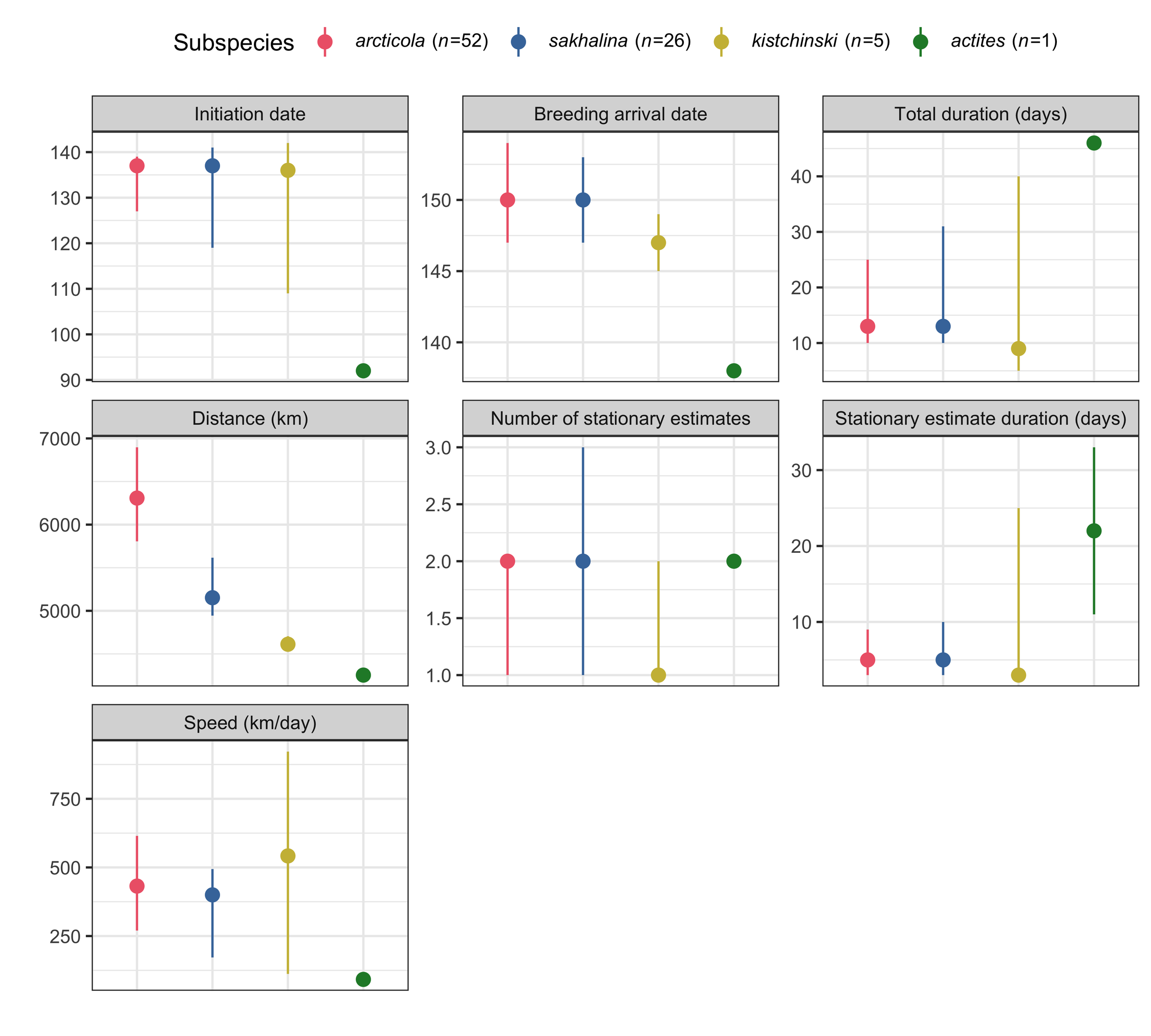

Supplement: S3 Fig — Reported is the median value and interquartile range. (TIF) [file pone.0270957.s006.tif]
