# Supplementary material for: Migratory network reveals unique spatial-temporal migration dynamics of Dunlin subspecies along the East Asian-Australasian Flyway
Source: PLoS One. 2022 Aug 4;17(8):e0270957. doi: 10.1371/journal.pone.0270957 (PMC9352067; doi:10.1371/journal.pone.0270957)
Supplement: Attachment — Submitted filename: Response to Reviewers.pdf [file pone.0270957.s007.pdf]

Reviewer #1: Thanks for sending me this interesting manuscript PONE-D-22-05779 by Benjamin Lagasse et al. titled "Flyway network model reveals unique spatial-temporal migration dynamics of Dunlin subspecies along the East Asian-Australasian Flyway". I am glad to see the data from this amazing long-term collaborative tracking effort being analyzed and written up. The authors summarized the migration characteristics of four Dunlin subspecies along the flyway. Despite being a common species along the flyway, we know very little about the migration pattern among different subspecies, especially when observing them in the non-breeding grounds. This study also provides important insights in monitoring and conservation effort and I added a few comments below which could be used to improve the manuscript.

General comments:

1. I agree with the authors that this work is useful for guiding on-the-ground survey efforts. It would be even more useful if the authors can be more specific here, either in the main text or as supplementary materials, to state when is the good time for a particular region to conduct its Dunlin survey (probably the time when only one subspecies occur?) to achieve long-term monitoring goals.

We now include a supplementary table detailing south migration timing of Dunlin subspecies by migration region (regions 14–25).

2. Line 468 – does this mean no sakhalina occured in the two regions? It's unclear if this percentage represent the subspecies composition within one region, or regional composition of one subspecies. I believe it's the latter, and if that's the case, then the presentation using region as the subject in a sentence to report the percentage is confusing (Sorry if I get this wrong). The same applies to the rest of this paragraph, it would help to clarify this at the beginning of this paragraph.

We revised the paragraph for clarity.

3. Line 565 – The departure date seems to be different to some published records (https://cms.hkbws.org.hk/cms/component/phocadownload/category/25-reports-of-shorebird-monitoring) and my personal observation in southern China such as Leizhou Peninsula. Dunlin in southern China 'disappear' in March or even earlier. It would be good to mention this phenomenon and the potential reasons behind the difference in those records with this geolocator study. Base on the definitions used in this study and the resolution of geolocator data, could a short move from southern China to the Yellow Sea be captured in the analysis?

A short move from southern China toward the Yellow Sea (particularly, if along a N-S axis) may not be captured in the analysis due to the coarse resolution of the geolocator data.

3 Dunlin (Sakh_d896, Sakh_6189, Acti_2295) had location estimates near the Leizhou Peninsula. We estimated they departed the Leizhou Peninsula area on 1 March, 10 March, and 2 April, respectively. After departing the Leizhou Peninsula area, we estimated Sakh_d896 and Sakh_6189 stopped for > 42 days before proceeding to their breeding sites (S1 Dataset). According to the criteria used for assigning north migration initiation dates, we estimated

Sakh_d896 and Sakh_6189 initiated north migration on 18 May and 18 May, respectively. We estimated Acti_2295 initiated north migration on 2 April.

We find Figure 3 sufficiently describes the arrival and departure timing of Dunlin subspecies by winter region (regions 1–13).

4. Lines 633-635 – note that this seems to contradict to what was said Lines 484-487. Sorry if I misunderstood something here.

We don't find a contradiction. Lines 484-487 state that individuals migrating north typically traveled faster, and lines 633-635 state that individuals migrating south typically traveled slower, etcetera.

Specific comments:

Lines 77: This sentence appears in the manuscript several times but please consider reword it a bit to explain clearly what was meant to say. An individual can 'winter' only in one place isnt'? Perhaps 'No individual Dunlin demonstrated movement between ... within the same winter'?

According to the criteria we used for assigning individuals' migration and wintering periods, individuals may winter in more than 1 location.

Lines 104, 109: Consider 'population decline'

We now include "population decline"

Line 131: instead of 'migration sites', consider 'stopping and wintering sites'

We now include "stopping and wintering sites"

Line 132: Consider citing Zhang et al's work, which showed a different way of degradation than the three references cited (doi:10.1017/S0959270917000430)

We now cite Zhang et al. 2018

Line 140: please also report the rates

We now report the rates

Line 142: instead of M. Tian pers. comm, now there is published record for this "a 28.4% decline in the Chinese Yellow Sea within about a decade" (doi.org/10.1016/j.biocon.2022.109547)

We now cite Wang et al. 2022.

Line 171: is there a name for this pattern? Parallel migration?

We revised the sentence for clarity.

Line 178: Should reword "which breed between the 2 other subspecies" as this sounds a bit strange.

We revised the sentence for clarity.

Lines 533, 534: Perhaps ‘times’ instead of ‘timing’?

We revised the sentence for clarity.

These comments have been very useful. We appreciate the time it took to provide this review.

Reviewer #2: First, I would like to congratulate the authors presenting a comprehensive analysis of the migration routes and timing and wintering movements of several subspecies of Dunlins in the East Asian-Australasian Flyway (EAAF).

This is a key contribution towards conservation of this species in the EAAF. One thing that really puzzles me is the title and framing of the manuscript as presenting a 'flyway network model', since I could not find any network analyses being presented in the manuscript. For a manuscript about a network model, I would expect the calculation of network metrices such as relative node strength and betweenness centrality (see Jacoby and Freeman 2016 for a good overview of methods applicable in movement ecology). In the second paragraph of the Introduction, several papers that applied network theory to evaluate patterns of migratory connectivity, identifying important sites, etc. were cited. Therefore, I am surprised not finding the analyses.

Unless the authors would like to conduct additional analyses based on network theory, I would suggest framing the manuscript differently. The results presented are valuable to understand the migration ecology of this species and highly informative in its conservation. I suggest deleting the 'flyway network model' in the title. For example, 'Migration patterns of four Dunlin subspecies along the East Asian-Australasian Flyway' would be a more appropriate title. Accordingly, the Introduction would need to be heavily revised, as well as other parts of the manuscript.

We revised the title. We also replaced 'flyway network model' with 'migratory network,' throughout. These changes are aligned with a similar study on Far Eastern Curlew that was recently published in Conservation Science and Practice.

Morrick ZN, Lilleyman A, Fuller RA, Bush R, Coleman JT, Garnett ST, et al. Differential population trends align with migratory connectivity in an endangered shorebird. 2022. Conservation Science and Practice. 2022; 4:e594.

Further comments:

Line 72: delete 'to construct a flyway network model' and rewrite

See above.

Line 104: I don't get why 'however' is used. There are some studies that focuses on spatial connectivity, but there are also a lot of studies about both spatial and temporal aspects of migration.

We revised the paragraph for clarity.

Line 126: The subtitle 'study system' in the Introduction is not necessary.

We find including the subtitle "study system" helps to orient the reader.

Line 154: It is good to mention results of Lagasse et al. (2020a), however too many details are included. This paragraph can be shortened substantially and combined with the previous one. For example, you can try to summarise it in one sentence starting with, 'Recent analysis of band recoveries reveals…'

We find the details in this paragraph provide important background information and identify the knowledge gaps this study aims to fill.

Line 166: I think there needs to be more elaboration on why knowledge on migration patterns is informative in developing flyway conservation actions. You can introduce the importance of identifying stopover sites here, as this is a main result of the manuscript but not much is said about it in the Introduction.

We now elaborate on how a lack of knowledge of migration patterns impedes the development of flyway conservation actions.

Line 169: The authors should rethink what is the main aim and objectives of this manuscript and revise this paragraph accordingly.

We have contemplated the main aim and objectives of this manuscript. We revised the paragraph for clarity.

Line 263: Instead of stating that Dunlin is a 'terrestrial species', it is better to specify that Dunlins cannot rest or forage in deep water.

We now specify that Dunlins cannot rest or forage in deep water.

Line 267: 'same for all Dunlin': say 'same for all individuals' instead.

We now say, "same for all individuals."

Line 271: which R package is this 'stationary.migration.summary' function from?

The packages are stated at the beginning of the previous paragraph.

Line 273: Please elaborate the biological meaning of 'stationary estimates', either here or at another part of the manuscript. This is used a lot in the Results and it will help interpretation of your findings to link it to more common terms, such as a stopover site or staging site.

We now elaborate on the biological meaning of "stationary estimates."

Line 298-300: I don't understand this sentence, what exactly are you trading-off?

We agree this sentence is confusing, and we find the paragraph isn't necessary, so we removed it. The previous paragraph provides the necessary justification for our calibration approach.

Line 314: I do not understand this procedure. Why do you discard stationary estimates that had a turning angle of <60 degrees? If you assume an individual migrated without reversing direction (line 319), then you should discard points that have a large turning angle instead? I could not find this procedure described in the reference given - Edelhoff et al. 2016 (line 316). I wonder how discarding these stationary estimates would affect the resulting flyway regions.

See S1 Appendix for a more detailed description of the procedure.

See Table 1 of Edelhoff et al. 2016, and the references therein, for more information on turning angle.

We discarded these stationary estimates, "…because FLightR can generate erratic stationary estimates due to noisy light-level data…"

Discarding erratic stationary estimates likely improves the identification of biologically relevant flyway regions.

Line 363: It is hard to understand the phrase 'to avoid potentially misinterpreting differences in migration speed between individuals' – what types of misinterpretation are you avoiding? I think it made sense to look at withinindividual changes in speed between north and south migration, maybe just rewrite the reasoning.

The misinterpretation is outlined in the previous sentence (i.e., attributing differences in speed between individuals to differences in migratory strategy, whereas differences might be due to differences in fueling demands).

We agree, it makes sense to look at within-individual changes in speed between north and south migration.

Line 368: I do not think the analyses described here is about constructing a flyway network model. It is just a method to cluster the stationary estimates.

See above.

Line 375: In this paragraph, you described how 'flyway region' is extracted, but 'flyway region' is an uncommon term and you should explain what it means in terms of stopover ecology. I imagine it could be a cluster of stopover sites since a 'normal' stopover site would have a much smaller diameter.

We find that what "flyway region" means in terms of stopover ecology (i.e., a cluster of stationary estimates) is explained in the paragraph (e.g., sentences 1 and 3).

Line 413: do you know the sex of this C. a. actites individual? Perhaps it is the sex that takes care of the young. The timing differences between the other three populations that you found could also be due to differences in breeding success and the sexes that were tracked. Do you know the sex of the birds and whether some tagged birds had nest failures? Failed breeders might leave for

southward migration earlier. I think an analysis that takes these two factors into account is necessary to really say something about differences between the subspecies. Hatching success should be able to be determined from the geolocator data by extracting the incubation period (e.g. Verhoeven et al. 2020).

Paragraph 1 of the methods section states:

"Adults were captured at nests during incubation or while attending broods using a bow net or mist net, respectively (Priklonsky 1960)."

"Geolocators were placed on both adults of a breeding pair, when possible, to maintain an even ratio of males and females within our tagged populations. Adults were also measured (wing, tarsometatarsus, total head, culmen) and had feathers and/or blood collected for archival purposes (Brown et al. 2014)."

Possible effects of sex and breeding success:

3 C. a. sakhalina were captured while attending broods. All other birds were captured while incubating eggs. We attempted to control for the possible effects of sex and breeding success by placing geolocators on both adults of a breeding pair, and by placing geolocators on multiple breeding pairs at each site.

Male and female Dunlin overlap in morphometrics. Given the limits of our data, molecular analyses are required to definitively assign individuals to sex. We have not conducted the molecular analyses necessary to definitively assign individuals to sex. We do not know the sex ratio of the birds recaptured. However, given we used similar deployment protocols across sites, it is possible sex ratios are similar across subspecies.

Failed breeders and female Dunlin may depart the breeding grounds earlier. However, whether they depart earlier may depend on where and when individuals undergo primary molt. For example, C. a. arcticola molt on the breeding grounds and, therefore, departure timing may depend on when individuals complete their primary molt rather than on their breeding success. Of the C. a. arcticola for which we have nest fate observations (n=53), individuals departed on similar dates regardless of their breeding success (median south migration initiation date and interquartile range; hatched = 2 Sep, 24 Aug–8 Sep, n=34; failed = 28 Aug, 22 Aug–10 Sep, n=19). The same is true for C. a. sakhalina (hatched = 13 Aug, 12 Aug–23 Aug, n=13; failed = 23 Aug, n=1). We do not have nest fate observations for C. a. kistchinski.

Fig 2: This is not a flyway network model. It does not describe the relationships between the flyway regions statistically or mathematically (e.g. based on graph theory) other than plotting a line between them. The caption would be fine if you start with 'General (A) south migration…'. Also, the part 'migrating and wintering birds' in the sentence 'see methods for determining migration and wintering regions…' seems redundant. Among figure 2A to D, I find only figure 2D is informative. The circles/triangles/squares in Fig A to C are quite misleading as the actual stationary estimates that each shape represents covers a much larger area, according to Fig

2D. Instead of these diagrams, I would really like to see the migration tracks used by different subspecies of Dunlin plotted with a different colour to show the differences and overlaps between subspecies' migration routes (the colours can be slightly transparent to show overlaps). Such a map will illustrate what you described in line 512-519.

We agree that Figure 2 does not describe the relationships between the flyway regions statistically or mathematically, and therefore, is not a flyway network model.

We revised Figure 2.

Line 497: To be consistent with the wording, should it be 'flyway regions' instead of 'migration regions'?

We agree this is confusing. We now define "migration regions" at the beginning of paragraph 3 of the results section.

Line 532: do you have any information on migration phenology of Peregrine Falcons in the EAAF? If not, there is really no support for this guess. Even Lank et al. (2003) have presented alternative hypotheses that are not danger-based. I suggest changing this sentence to reflect that there are several hypotheses (not mutually exclusive) that can explain these patterns.

We state:

A) *We expected this pattern to result from earlier breeding phenology at lower latitudes*

B) *it may also reflect differing strategies for how subspecies minimize their exposure to predation by Peregrine Falcons (Falco peregrinus)*

C) *Alternatively, subspecific differences in south migration timing may reflect differing strategies for where and when subspecies undergo flight feather molt*

We provide several hypotheses. We now cite a source that describes the migration phenology of Peregrine Falcons.

Line 574-578: The logic of this sentence does not make sense. Your results agree with the general predictions that migrants used a 'time minimization strategy' during northward migration and 'energy minimization strategy' during southward migration (Alerstam and Lindström 1990). But this strategy itself does not lead to optimal timing of arrival, which is achieved by migrating at the right time, e.g. by using environmental cues that predict breeding ground food availabilities. Also, the strategy does not lead to higher stress levels. The papers you cited at line 577-578 are about species that are facing habitat degradation during northward migration. These negative impacts are not expected in situations with good stopover habitat quality.

We agree and appreciate being made aware that the sentence does not make sense. We removed the sentence from the manuscript.

Line 581: In this paragraph you should also discuss that the southward migration timing could be a result of breeding success and the sex of the individuals tracked.

See above.

Line 612: if the subspecies are not distinguishable from each other during field observations, it seems impossible to estimate, from flock counts and tracking data, the number of individuals of each subsepecies that use a particular site. To achieve that would also require knowing the population size of each subspecies and the proportion of the population that uses a particular stopover site. The latter might be able to be derive from tracking data, but that would require a very large sample size.

We found adult Dunlin migrated south in temporally distinct waves. We suggest adult Dunlin observed during south migration may be assigned to subspecies according to where and when they are observed.

We now provide a supplementary table detailing south migration timing of Dunlin subspecies by migration region (S1 Table).

Line 614, 639: To estimate population size and trends, the obvious method would be counting at wintering regions. I wonder how that could be done at stopover sites (as suggested in these lines) where several subspecies co-occur and you cannot visually distinguish them from each other. If you have a valid method in mind, please elaborate more.

See above.

Line 638: It would be better to specify which part of the results is useful in guiding on-the-ground survey efforts. I think spatially it is not really useful as the area indicated (including the uncertainty of 200 km) is way too large. Only the timing of when Dunlins occur at a specific area would be useful. From the point of view of somebody doing ground surveys, it is quite difficult to derive the timing from Fig. 4. It will be much easier if the range of timing is provided in a supplementary table.

We now specify which part of the results is useful in guiding on-the-ground survey efforts.

We now provide a supplementary table detailing south migration timing of Dunlin subspecies by migration region (S1 Table).

References:
Alerstam, T., and Å. Lindström. 1990. Optimal Bird Migration: The Relative Importance of Time, Energy, and Safety. In Bird Migration, 331–51. Springer Berlin Heidelberg.

Jacoby, D. M., & Freeman, R. 2016. Emerging network-based tools in movement ecology. Trends in Ecology & Evolution, 31(4), 301-314.

Lank, David B., Robert W. Butler, John Ireland, and Ronald C. Ydenberg. 2003. Effects of Predation Danger on Migration Strategies of Sandpipers. Oikos 103 (2): 303–19.

Verhoeven, M.A., Loonstra, A.H.J., McBride, A.D., Macias, P., Kaspersma, W., Hooijmeijer, J.C.E.W., van der Velde, E., Both, C., Senner, N.R. and Piersma, T. (2020), Geolocators lead to better measures of timing and renesting in black-tailed godwits and reveal the bias of traditional observational methods. J Avian Biol, 51

These comments have been very useful. We appreciate the time it took to provide this review.

Reviewer #3: In their study, Lagasse and colleagues provide a flyway network model based on tracking data from different breeding populations and subspecies of Dunlins migration through the East Asian-Australasian Flyway. Using the network approach, the authors characterized migration strategies (routes, phenology) and compared the different breeding populations and subspecies. First, I would like to congratulate all authors on collecting such an impressive collection of tracks from this species and for putting them together allowing to compare the subspecies and reveal some of the larger scale patterns of migration in the EAAF. The paper is very well written and easy to understand. The methods include one of the most elaborate and transparent description of light level geolocation I have seen and the work the authors put into the track estimates allowing robust conclusions. While reading the paper, I of course though that information on population dynamics would make the study so much more valuable. I am aware that that information is not yet available (and that there are plans already installed in will likely be installed in the future to get these urgently needed information). The lack of these data is discussed in the "Limitations and future directions" section, and I am sure that the network developed in this study can be used on the future and may even can inform effective monitoring plans.

In my opinion, the paper is ready to be published. If I can suggest a slight improvement, it would be to also put some of the values presented in the tables into figures (e.g. departure/arrival/wintering duration etc. for the different populations). I think that this would make it easier to see the differences/communalities. I got a (tiny) but frustrated while trying to extract and understand the dates/periods and picture them myself in the head. I ended up visualizing them for myself to get a better impression.

We now include supplementary figures depicting Tables 2–4.

Two additional very minor comments:

L95: Sounds like migration only presents risks. Maybe add that migrations evolved due to the opportunity to exploit optimal conditions across regions.

We find that focusing on risk provides a more concise introduction to the purpose and significance of the study.

L124: What about Morrick et al. 2022 Conservation Science and Practice

We appreciate being made aware of this recent publication. We removed the last sentence of the paragraph.

Again, congratulations on the data collection and the synthesizing analysis across populations. I am sure that this study is of interest to the migration/shorebird community and that the methods described will be appreciated and repeated in future studies.

These comments have been very useful. We appreciate the time it took to provide this review.
